# A Review of emergency medical services for stroke

**DOI:** 10.4314/ahs.v24i3.42

**Published:** 2024-09

**Authors:** Yanxia Wu, Ken Li, Lizhen Tang, Guang Li, Dongxue Huang, Yahui Yang, Shihui Song, Li Peng

**Affiliations:** 1 Department of Emergency, The Second Affiliated Hospital of Xingtai Medical College, Xingtai, China; 2 Department of Neurology, Baoding NO.1 Central Hospital, Baoding, China; 3 Department of General Surgery, Chengde Central Hospital, The Second Clinical, Chengde, China; 4 Department of Emergency, Chengde Central Hospital, The Second Clinical, Chengde, China; 5 Department of Neurology, Chengde Central Hospital, The Second Clinical, Chengde, China

**Keywords:** Emergency medical service, stroke, management, strategies, technologies

## Abstract

In the past decade, Emergency Medical Services have been associated with innovations in technology; the 911 telephone system and two-way radio have developed the notification, scheduling, and response processes. The recent twenty years have witnessed the unparalleled innovation changes of the computer framework. These new frameworks in mobile, social, cloud computing or big data concentrations essentially affect the entire society. In the last ten years, major innovation and strategic improvements have occurred, which will affect the concepts and communication methods of Emergency Medical Service in the future. Emergency Medical Service can treat various diseases in the correct way. For example, Emergency Medical Service personnel's early recognition of stroke performance is an important ideal consideration for patients with stroke patients. Pre-stroke screening tools that have been preliminarily evaluated for sensitivity and specificity are necessary to improve detection rates for the pre-court stroke by Emergency Medical Service experts. This is an excellent time for Emergency Medical Service to play a key role in achieving and transcending vision. The motivation behind this article is to provide extensive investigations and unique opportunities for Emergency Medical Service personnel groups to solve how to improve.

## Introduction

Innovative technology has long been associated with Emergency Medical Service (EMS); 911 phones and two-way radios provided the foundation for the first notice, dispatch, and response frameworks. Recent decades have witnessed an unprecedented innovation upheaval in computerized systems, including the numerous ways and structures that data can be prepared, stored and imparted. These new frameworks in portable, social, cloud-based, or bigdata concentrated are essentially influencing businesses all through society. In the days before the advent of hearty, remote broadband organizations, cutting-edge versatile processing capacities, and profound information insight abilities, it is impossible to know for certain how EMS would have been structured strategically, authoritatively, or financially. EMS may have to be re-examined, updated, upgraded, and retrofitted to keep up with innovations as they progress^1^. EMS might play a vital role in certain conditions, for instance stroke patients get right medical attention by EMS, instead of waiting for emergency department attention^2^. Furthermore, EMS initiation and transport is emphatically connected with stroke onset and arrival to emergency department. Starting from emergency medicine physician evaluation, computed tomography (CT) imaging, and evaluation of neurologist^3^. Present treatment rules by the American Heart Affiliation (AHA) and American Stroke Affiliation (ASA) suggest prehospital notice may elevate the stroke patient care by EMS^4^. It also improves the productivity and conveyance of stroke care taking into consideration of hospital personnel and resources, thereby reducing the evaluation time, testing, and treatment^5^. This is a perfect time for EMS to assume a critical part in accomplishing and outperforming the vision. EMS experts should now address how EMS acts on stroke management to make the most of the accessible chances. It is the purpose of this write-up to provide a comprehensive survey and distinctive evidence of opportunities for the EMS group to address innovation improvements in stroke management.

## Prehospital stroke assessment by EMS and the pre-hospital management of stroke

Early recognition of stroke manifestations by EMS personnel is an important piece of ideal consideration for casualties with stroke. The use of prehospital stroke screening instruments that have been tentatively assessed with sensitivity and specificity^6^ will improve stroke detection in prehospital settings^6^. The accuracy by paramedic to identify stroke patients by screening is somewhere between the range of 61% and 72%^7^.

The usage of prehospital stroke evaluation instrument has been growing among the paramedic these days. Important basic prehospital stroke assessment instruments that are being widely used are the Los Angeles Prehospital Stroke Screen( LAPSS) and the Cincinnati Prehospital Stroke Scale (CPSS) as these two instruments shows about 90% of accuracy when assessing individuals for stroke. Furthermore, Melbourne Emergency vehicle Stroke Screen (MASS), which is a combination of the CPSS and LAPSS, has additionally demonstrated an accuracy about 90%^8^. A stroke detection instrument that is adjusted for EMS personnel may be viable for improving stroke care, according to some studies^9^. EMS faculty ought to be comfortable with a strategy of prehospital stroke evaluation and use it regularly on patients associated with stroke. Some studies suggest that stroke detection instruments adjusted to EMS personnel can improve stroke care^10^.

Regardless of the significance given in identification of stroke. The stroke-related topic has been given less consideration in the transportation^11^. Finally, to provide a better care every EMS personnel ought to have explicit fundamental information and must be able to manage the patients by following proper stroke assessment guidelines for better treatment outcome^12^.

Pre-hospital management of stroke refers to the immediate care and interventions that are provided to a person suspected of having a stroke before they are transported to a hospital. This is a critical time for stroke patients as the prompt initiation of appropriate treatment can significantly improve their outcomes. Here are some insights into the pre-hospital management of stroke:

1. Early recognition: The first step in pre-hospital management of stroke is to recognize the symptoms of stroke as early as possible. The common symptoms of stroke include sudden weakness or numbness on one side of the body, slurred speech, difficulty in speaking or understanding speech, sudden vision problems, dizziness, and severe headache.
2. Activating emergency medical services (EMS): Once stroke is suspected, it is important to call for emergency medical services (EMS) immediately. In many countries, the emergency number is 911. The EMS team will assess the patient's condition and provide appropriate treatment, such as oxygen and medications to control blood pressure, if necessary.
3. Transport to a stroke center: The EMS team will transport the patient to a hospital that has a stroke center as quickly as possible. A stroke center is a specialized facility that has the resources and expertise to provide advanced stroke care, including the administration of thrombolytic therapy or mechanical thrombectomy.
4. Assessment and treatment: During the transportation, the EMS team will continuously monitor the patient's vital signs and perform further assessments to determine the severity of the stroke. If the patient is stable, they may receive further treatments such as oxygen, glucose, or aspirin to prevent complications.
5. Communication with the hospital: The EMS team will communicate with the hospital to provide information about the patient's condition, symptoms, and medical history. This helps the hospital team prepare for the patient's arrival and provide appropriate care, the pre-hospital management of stroke is a crucial step in providing timely and effective care to stroke patients. Early recognition, prompt activation of EMS, and transportation to a stroke center can significantly improve the patient's outcome.

## Role of EMS in stroke

Stroke survivors need to save their brains and reduce mortality and bleakness as soon as possible 13. The fundamental factor restricting the utilization of specialists for ischemic stroke is the three-hour window for recombinant tissue plasminogen activator (rt-PA). Reperfusion treatment may be effective in some cases when directed up to 4.5 hours after the onset of symptoms^2^, or even up to 6 hours after onset. A basic factor for consistence with these occasions is the assistance of fast home-to-clinic moves^14^. Stroke assessment is possible while the casualty is transported by EMS than in instances of self-transportation to the emergency clinic. When administered up to 2 hours after the onset of symptoms, or even up to 6 hours later, reperfusion may be effective in some cases^15^. The majority of patients failed to arrive at the medical clinic in time, resulting in a smaller number of patients being treated with Rtpa^16^. Efforts are needed to be made to diminish transportation times^17^. Based on investigation, time between the brain imaging was essentially more limited in instances of EMS transportation, so patients calling EMS had opportune imaging of brain in 2.7 occasions more frequently than patients utilizing self-transportation. This outcome applies to the standard time of 2 hours just as to the all-inclusive season of 3 hours 30 minutes seen in some cases^18^. Patients with stroke indications, who decided to call EMS for emergency vehicle transportation had more extreme indications than patients utilizing self-transportation^14^. Though EMS is considered to be playing a major role on stroke patient evaluation and management there is a need for improvement in delivery with the help of new strategies, that can help to achieve desired treatment outcome.

## Strategies in stroke management

Few factors are significant in deciding a medical clinic's ability in giving enhanced stroke care. The significant components incorporate (1) the presence of doctors with ability (2) the accessibility of imaging (CT or X-ray) and sufficient auxiliary care, and (3) the accessibility of learned work force to complete affirmed stroke treatments including the utilization of IV- rt-PA, (4) the presence of an institutional plan to deal with, or possibly give beginning assessment of, essential haemorrhages and haemorrhagic change of infarcts^19^. < = “” span=“” style=“font-family: “Times New Roman”;”> has archived the improved result of patients who get in-emergency clinic care instead of primary clinics showed considerable stroke care.^52^–^54^ Furthermore, moving patients with quick admittance have elevated TPA use^20^.

## New technology-centric EMS education and training modalities

Suitable EMS preparations in advanced innovations is basic to its prosperity. This is valid at the operational level where EMS faculty need to incorporate advanced technologies into their work process in a way that upgrades and doesn't cheapen understanding consideration. It is likewise evident at the administration level, where EMS and medical care supervisors need to incorporate such frameworks into management information systems (MIS). For instance, “chief information officer (CIO)” gatherings talk about the most recent patterns, advancements, and best practices for utilizing innovation to take care of enormous issues. Clinical and analytic gadgets that are information empowered for example; ultrasound additionally need consideration^21^. These gadgets are not regularly joined into preparing projects and accordingly a cycle is expected to figure out which gadgets ought to be educated and when. The gadgets referred to here are not typically integrated into standard training programs, which means that an additional process is necessary to determine which devices should be included in the training curriculum and when they should be taught. This process requires careful consideration of factors such as the complexity of the gadget, the level of proficiency required by the trainee, and the relevance of the gadget to the overall training objectives. Moreover, without a formal process in place to incorporate these gadgets into the training program, trainees may miss out on important skills and knowledge that could be gained from using them. It is essential to identify the most suitable gadgets and integrate them into the training curriculum effectively, as this can enhance the trainee's experience and ensure that they are fully prepared to use the technology in their future work. Therefore, it is crucial for organizations to evaluate and regularly update their training programs to include the latest technologies and gadgets that are relevant to their industry. Affirmation measures are required for utilizing new gadgets in EMS. A critical test is that totally new advances need outside master schooling as opposed Along these lines, more conventional schooling is needed for educating and guaranteeing competency in innovation subordinate systems and cycles^1^. To conclude there is a need to exploit innovation empowered learning frame-works^22^. The possibility of utilizing innovation has been explored^23^. EMS staff (and patient consideration) could profit by getting information and abilities about quite certain techniques, conventions, approaches, and other substance at basic crossroads focuses^24^. It will be good to have a better understanding among the medical world to provide a better collaborative approach in delivering a better treatment needs by using the latest technologies and innovations.

Recent advancement tools and strategy of EMS service Magen David Adam (MDA) is the EMS supplier for Israel. It has been formally perceived by the 40 000 volunteer's subsidiary with MDA (About Israel in 2018 population; 8.4 million). The volunteer foundation is on a public level, is multitiered in plan, and segregated into a few divisions. Divisions incorporate a blend of volunteers who basically fill in as staff on ambulances and an assortment of on call volunteer first response (OCVFR)^25^.

The emergency vehicle-based volunteers incorporate customary grown-up volunteers at the degree of emergency medical technicians, yet in addition nearby secondary school students and school age global volunteers. The OCVFR incorporate the individuals who react from work or home put away in their vehicle, also as a unique gathering of bike-based volunteers. There is another group of Life Guardian people on call who are generally medical care laborers who can manage myocardial infarction^26^.

MDA utilizes a few telecom techniques to initiate the volunteers. Every gadget has its own exceptional preferred position, and all demonstration in show for a productive and fast reaction. MDA utilizes a division of more than 50 people, including programming software engineers, support staff, network safety trained professional, data innovation specialists^27^.

## EMS telecommunication tools

### One way pager

The 1-way pager is a remote broadcast communications gadget that gets and shows alphanumeric messages. It is a mass public ready and cautioning framework, which is one of the quickest and the solitary mass alarm offering available today that can send a solitary message to as many gadgets all the while in less than 20 seconds^27^.

### Push-to-talk over cellular

The gadget's diminishing prominence empowers the utilization of this innovation even in myocardial infarctions when different wireless transmissions might be in substantial use. In spite of the pager, PoC has a 2-way highlight, and its Global Positioning System (GPS) empowers the accurate location of the call placed^27^.

### Smartphone application

The mobile application developed by MDA naturally distinguishes all volunteers who are in vicinity and ready to show up at the scene within 5 minutes, just as the 8 nearest chips in at a more noteworthy distance. The application initiates the volunteers just in instances of crisis, for example, patients in heart failure or survivors of injury and patients with stroke^27^.

### Stroke analysis scales

There are numerous stroke scales available for prehospital use that have been published recently^28^,^29^.

### National institute of health stroke scale (NIHSS)

The new NIHSS was developed and validated clinically as the first scale for all-in-one rapid and comprehensive prehospital stroke assessment and stroke recognition which can be used to evaluate parallel stroke recognition, severity grading and Large vessel occlusion (LVO) prediction. The use of multiple stroke scales for EMS must be avoided^30^.

The test characteristics for patient identification with LVO are non-inferior to existing LVO prediction scales. However, item assessment compatibility with full-length NIHSS permits precise assessment of the clinical path from pre-hospital to in-patient care. Hence, it is considered as the ideal stroke scale for regular use in pre-hospital EMS.

Sensitivity of the NIHSS-EMS regarding stroke recognition was higher i.e. (91 %) than other scales such as Cincinnati Prehospital Stroke Scale (CPSS; 85%) and Field Assessment Stroke Triage (FAST; 87%) assessed previously.

The specificity of the NIHSS-EMS was lower i.e. (52%) compared with the CPSS (65%) and FAST (64%). The overall load of a missed stroke outweighs the potentially increased burden for emergency departments. Greater sensitivity may be regarded as more significant. Simple stroke scales may impart a faster assessment initially but requires another scale for assessing the severity of stroke or LVO prediction. The sNIHSS-EMS is another comprehensive scale compatible with NIHSS.

### RACE scale

The Rapid Arterial Occlusion Evaluation (RACE) is a high accuracy global scale which was obtained with the addition of 5 items that ultimately built the RACE assessment scale for facial palsy, arm motor function, leg motor function, gaze, and aphasia or agnosia^31^,^32^. The RACE scale assessment has high sensitivity rate of (85%) and specificity rate (65%) to detect LVO.

The prospective validation of the RACE scale involves the Stroke Code (SC) protocol. SC system has been functional from past eight years and it is activated by EMS or community hospital sectors concerning any patient with an acute stroke of clinical suspicion with an onset 6 hours from moderate to severe symptoms. There are about 60% or more patients with acute stroke were brought to the hospital usually transferred by basic or advance emergency care ambulance services. Hence, this scale is the first validated tool to identify the patients with LVO and acute stroke at a prehospital set up^33^.

### Cincinnati pre-hospital stroke scale (CPSS)

The CPSS is routinely used for detecting prehospital stroke. This scale can be used to evaluate the LVO stroke in unselected patients with acute neurological signs in a prehospital set up and to determine the effect of scale scoring strategy on patient volume, treatment time, and clinical results. The CPSS scale was modified in the year 2016 in which high CPSS score indicates positive for all three items (3/3) and would serve as an excellent method to identify LVO in the prehospital set up thereby decreasing the resources required for the administration of a new stroke severity scale. Correspondingly, any patient with a greater CPSS score (3/3) and the last onset symptom well before five hours was transported directly to the nearest Comprehensive Stroke Center (CSC) thereby avoiding Primary Stroke Center (PSC) and EMS Redirection was implemented in October 2016. By utilizing EMS redirection protocol with high CPSS score (3/3) enhances the detection of LVO stroke in out-of-hospital set up^34^. The sensitivity and specificity of CPSS scale for stroke assessment were reported to be higher i.e., 94% and 20% respectively when compared with other scales^35^.

EMS protocols, which refer patients with suspicion of stroke-to-stroke centers mainly depends on the application of precise screening criteria for stroke. The CPSS and the Los Angeles Prehospital Stroke Screen (LAPSS) scales are most commonly used screening instruments for stroke.

### LAPSS

LAPSS and CPSS are similar in test characteristics, the sensitivity and specificity of both the scales are also same. The poor specificity can lead to “Overtriage,” with most of the non- stroke patients being routed to stroke centers and specificity presumes specific significance when stroke screens are being utilized for transport diversion. Some level of Overtriage is justified in order to improve overall access to thrombolytics and other therapies of acute stroke, the following question arises, what rate of Overtriage is admissible within a statewide system of acute stroke care? However, no Overtriage or Undertriage guidelines have been suggested so far for stroke systems of care^36^. In regionalized trauma care, the American College of Surgeons Committee on trauma care has recommended that an Overtriage rate of 30-50% is acceptable. Overtriaging can indeed improve access to timely treatment for patients who might otherwise miss out. However, it also carries inherent risks that must be taken into consideration. One of the main risks of overtriaging is the potential for harm to patients who receive thrombolysis when they do not meet the standard criteria for treatment. Thrombolysis carries a risk of bleeding, and patients who are treated without meeting the appropriate criteria are at higher risk for adverse events such as intracranial hemorrhage. Another risk associated with overtriaging is the potential for strain on healthcare resources. When more patients are identified as potential candidates for thrombolysis, there is a greater demand for resources such as emergency department staff, imaging facilities, and pharmacy supplies. This can lead to delays in treatment for other patients and increase the overall burden on the healthcare system. In case of trauma, priority for stroke must be given to Undertriage in order to reduce morbidity rate due to delays in proper care, but this may result in an extraneous financial and human resource which can bestow to crowding of stroke center, and can extend EMS transport and hospital processing times. Besides, non-stroke patients may bring to the hospitals within which their electronic medical record history would be difficult to access and their emergency physicians do not have privileges, thus contributing to, rather than decreasing, the fragment of care. This can disappoint patients, their family members, and care givers. Additionally, whenever hospital route is undertaken in more rural settings with a limited number of ambulances, improper direction may result in the unavailability of an ambulance to transfer an acute myocardial infarct (MI) patient to a transluminal coronary angioplasty center.

In conclusion, careful understanding of the out-of-hospital stroke screens accuracy within the stroke care system is utmost important. Fairly high sensitivity permits out-of-hospital providers to properly divert patients to stroke centers so that they can be alert or initiate resources while reducing the likelihood of bringing a stroke patient to a hospital who were unable to receive acute revascularization therapy^37^. The comparison of pre-hospital stroke scales has been mentioned in (Table 1). The sensitivity and specificity for pre-hospital LVO assessment using LAPSS were reported 0.43 and 0.88 respectively^38^.

**Table 1:** The comparison of pre-hospital stroke scales

Stroke scales	NIHSS	RACE	CPSS	LAPSS
No. of items scored	13	6	3	6
Sensitivity and specificity for stroke assessment	91% and 52%	85% and 65%	93% and 20%	74% and 48%
Sensitivity and specificity for LVO	69% and 85%	66% and 72%	85% and 65%	43% and 88%

## Advanced technologies

### Mobile stroke unit (MSU)

Mobile stroke unit is a rescue vehicle that outfits administrations to analyse, assess, as well as treat indications of an intense stroke condition. It might contain the typical rescue vehicle with a CT scanner, a state of-care lab and telemedical cooperation among rescue vehicle and emergency clinic with videoconferencing, recording exchange of patient assessment and CT investigation. This particular rescue vehicle incorporates all the instruments essential for hyperacute appraisal and treatment of stroke patients and assessment-based emergency straightforwardly at the crisis site^39^. For the issue of impeding postponements in stroke, Mobile Stroke Unit idea was created to identify treat the individual straightforwardly at the crisis site rather than anticipating the patient's landing in emergency clinic for treatment. By bringing imaging innovation and stroke clinical skill to the scene, the EMS personnel can exploit the pre-clinic appearance time by zeroing in collaborations exclusively on an individual with suspected stroke. At the emergency site, the MSU team is responsible for collecting a patient's case history, details about neurological and laboratory examination, a CT scan (where appropriate), and an assessment with the remote VN via telemedicine. If required, thrombolysis will be given onsite. As per the 2015 Canadian Stroke Best Practice Recommendations the management of acute ischemic stroke states that the standard of care for thrombolysis include t-PA and screening by the stroke physician. The t-PA must be given within 4.5 hours of the onset of symptoms and soon after the arrival at a hospital setting. For acute ischemic stroke the immediate decision to give endovascular therapy must be coordinated in a system that involves EMS; quick imaging; in-hospital arrangements between the department of emergency, radiology and a stroke team who are expertise in managing stroke patients^40^.

### Tele-stroke

Telemedicine has been characterized as ‘the utilization of media communications technology for clinical indicative, checking and helpful purposes when distance as well as time isolates the members^41^. The expansion of video to sound for the assessment of neurological patients, explicitly intense stroke, was first depicted in the mid 1990's, however the term tele stroke was instituted by Levine and Gorman for the real use of great intuitive telemedicine for intense stroke assessment and additionally intercession. The pair explained the utilization of video-telecom to interface vascular nervous system specialist's day in and day out to emergency physicians and their patients to give limitless admittance to emanant stroke treatment^42^. Startup expenses of a telemedicine network are enormous, for the most part because of the requirement of huge capital. These expenses can be additionally separated into the telemedicine gear, data innovation support, the important clinical and authoritative personnel, preparing and credentialing of work force, and funding for whole session if the need arises^43^. Yearly expenses are assessed at $46,000 yet can go from under $10,000 to more noteworthy than $200,000, depending upon office size, choice of technology and so on. These start-up costs are once in a while overwhelmed by research awards or public subsidizing, frequently in rustic or underserved regions. Accordingly, most of tele stroke networks are related with huge scholastic habitats in significant metropolitan territories serving rustic and far off regions^44^.

There are about 11 studies^45^-^59^ that assessed acceptability of tele-stroke systems from the viewpoint of EMS and remote clinicians utilizing combination techniques. Outcome was positive demonstrating high levels of satisfaction with tele-stroke systems with regard to high image quality, usability, reliability or considerable safety, while few studies suggesting only minor issues in relation to connectivity. In one study it has been reported that, only 25% of EMS nurses believed tele-stroke could improve emergency evaluations and decreased time-to-treatment due to the concern of clinician's ability to use tele-stroke systems and integrate into standard care procedures^60^.

### Prehospital imaging device

Prehospital ultrasound is a special kind of ultrasound by paramedics, to provide prompt consideration and treatment techniques. Like traditional ultrasound, it is a gadget that produces cyclic sound strain to enter flesh and uncover insights regarding the internal structure of the medium^61^. Prehospital ultrasound pictures muscle, delicate tissue, and bone surfaces well indeed and especially helpful for portraying the interfaces among strong and liquid occupied spaces, in contrast to most different strategies for injury determination, which are minimal more than taught guesses. It renders “live” pictures, where the administrator can progressively choose the most important segment for review, and limits the pain point, instead of holding up until the patient is at the emergency clinic. It doesn't cause any uneasiness to the patient. Sonographic device experience difficulty infiltrating bone. For instance, sonography of the adults is exceptionally restricted. This implies that regarding injury conclusion including mind injury, sonography will be troublesome and requires very good quality ultrasound machines^62^. The profundity infiltration of ultrasound is restricted, making it hard to picture structures somewhere down in the body, particularly in hefty patients. The strategy is administrator subordinate. An undeniable degree of expertise and experience is expected to procure great quality pictures and make precise judgments, which is one more ability that a restricted EMS group should create. Since most EMS groups are little and endure high turnover, holding qualified staff can be troublesome^63^.

Electroencephalography (EEG) provides detailed location in relation to cerebral tissue injury^64^. ‘Dry’ electrode cap EEG was used in the Electroencephalography controlled triage in the ambulance for acute ischemic stroke (ELECTRA-STROKE) study to diagnose anterior circulation of LVO for automated signal analysis in presumed stroke patients of cerebral ischemia in a prehospital setup. The technology is fully advanced; although, algorithm development is at preliminary stage. The EEG findings will only be analysed in the hospital setting so target selection will not be examined^65^.

The handheld Infrared device has also been studied for pre-hospital screening in USA by the trained EMS physicians while transportation. The main aim of using infrared device is to distinguish various types of strokes to find out variation in blood flow. The study involving hemorrhage versus mimic was evaluated, the authors reported sensitivity and specificity were 71% and 40% respectively. Another study evaluated hemorrhage versus ischemia was assessed in which sensitivity and specificity found was 71% and 43% respectively. However, all types of hemorrhagic strokes were identified by the infrared device but its poor specificity may restrict clinical use^66^.

### Biomarkers

Imaging biomarkers are progressively used to give a superior comprehension of the pathophysiology of intense ischemic stroke. However, this methodology of regularly utilizing imaging biomarkers to inform treatment choices still can't seem to be converted into effective randomized preliminaries. The point-of-care system consists of SMART-Chip, a disposable biosensor and a handheld reader (Sarissa Biomedical Ltd, UK.) which measures purines by a finger-prick blood sample technique. Purines accumulate rapidly during hypoxia (as in stroke) which are the by-products of cellular metabolism which can be detected in systemic arterial blood reliably, within 3–5 mins results will be obtained and paramedical staff requires training to operate this unit. n addition to purines, which can be used as markers for early stroke, there are also other biomarkers, the detail was shown as follows: S100B:

This protein is released into the bloodstream after damage to brain cells and can be used to detect stroke early. NSE (Neuron-specific enolase): Another protein that is released when brain cells are damaged, NSE levels can be used to indicate the severity of a stroke. GFAP (Glial fibrillary acidic protein): A protein found in the cells that support brain neurons, GFAP levels can be used to detect stroke and other brain injuries. BDNF (Brain-derived neurotrophic factor): This protein plays a role in the survival and growth of brain cells and has been found to be reduced in people who have had a stroke. MMP-9 (Matrix metalloproteinase-9): This enzyme is involved in the breakdown of the blood-brain barrier and can be used to detect stroke and other brain injuries. CRP (C-reactive protein): This protein is released in response to inflammation in the body and can be used to detect the risk of stroke. D-dimer: This protein is released when blood clots break down and can be used to detect the risk of stroke. But there are no portable devices that can be used for rapid pre-hospital testing. Currently there is not much literature about diagnostic accuracy or patient outcome data. Although, the technology is at a late stage of development which would facilitate further research^66^. Treatment choices for patients with intense ischemic stroke are restricted. Despite endeavours to create novel neuroprotectants and techniques for reperfusion, not many have made it into routine practice. This disappointment of progress is multifactorial in beginning; however, it incorporates the inability to appropriately represent persistent heterogeneity and an absence of demonstrated substitute outcomes^67^. Imaging has been generally embraced, both in clinical practice and researches, to accomplish different points, including: diminishing heterogeneity of members in a preliminary trial; defining patients into those that might profit by medicines; and, surveying mediation adequacy as well as safety^68^. The Acute Stroke Imaging Research Roadmap II was created on the foundation of late invalid acute stroke preliminaries incorporating the utilizing penumbral imaging. It fortified the requirement of significant interest, that characterize framework for utilizing imaging biomarkers in imaging study^69^.

## Conclusion

Emergency conditions like stroke happen suddenly to an individual. Emergency medical services incorporate quick evaluation, opportune arrangement of proper mediations, and brief transportation to the closest medical centres at right time. The objective of viable EMS is to give importance to an individual who require it^70^. Finally, it is recommended that there is a need for innovation, research and technological paths in EMS to provide a better care for the mankind. The consistent improvement in EMS depends only on the innovations and the future planning that might play a major role in delivery of Emergency Medical Services to every corner of the globe.

## Data Availability

The datasets used and analysed during the current study are available from the corresponding author on reasonable request.
